# Mechanical and Fatigue Properties of Welded Fe-Mn-Si Shape Memory Alloys

**DOI:** 10.3390/ma17174304

**Published:** 2024-08-30

**Authors:** Kinam Hong, Sangwon Ji, Dohyung Kim, Jinyoung Bae

**Affiliations:** 1 Department of Civil Engineering, Chungbuk National University, 1 Chungdae-ro, Seowon-gu, Cheongju 28644, Republic of Korea; hong@cbnu.ac.kr (K.H.); jinyoung5845@gmail.com (J.B.); 2 Dongnam Regional Division, Korea Institute of Industrial Technology, Yangsan 50623, Republic of Korea; dhyungkim@kitech.re.kr

**Keywords:** Fe-Mn-Si shape memory alloy, shape memory effect, welding fatigue characterization, high-cycle fatigue behavior, microstructure

## Abstract

This paper presents the experimental results of a study evaluating the mechanical and fatigue performance of welded Fe-Mn-Si SMA. For the experimental study, welded and welded-and-heat-treated Fe-Mn-Si SMA specimens were fabricated, and fatigue tests were performed at various stress amplitudes. In addition, direct tensile tests and recovery stress tests were also performed to evaluate the material properties of Fe-Mn-Si SMAs. The elastic modulus, yield strength, and tensile strength of the welded specimens were reduced by 35.4%, 12.1%, and 8.6%, respectively, compared to the values of the non-welded specimens. On the other hand, the elastic modulus, yield strength, and tensile strength of the welded-and-heat-treated Fe-Mn-Si SMA specimens were increased by 18.6%, 4.9%, and 1.3%, respectively, compared to the values of the welded specimens. Both welded and welded-and-heat-treated Fe-Mn-Si SMAs failed at lower cycles than the conventional Fe-Mn-Si SMAs at the same stress amplitude. High-cycle fatigue failure, characterized by cycles exceeding 10^4^, typically occurs at relatively low stress levels within the elastic region, whereas low-cycle fatigue failure, generally occurring within cycles below 10^4^, involves high stress levels that encompass both elastic and plastic deformation. Regardless of the welding condition, the stress amplitude at which Fe-Mn-Si SMA transitions from high-cycle to low-cycle failure exceeded the yield strength.

## 1. Introduction

Over the past few decades, shape memory alloys (SMAs) have attracted considerable attention due to their unique property of shape memory effect (SME), which allows them to recover their original shape at a certain temperature [1,2,3]. SME in SMAs is caused by the reverse transformation of martensite to austenite at high temperatures [4]. Therefore, SMAs can recover their original shape through phase transformation of the alloy in the plastically deformed state and can induce recovery stress, such as prestressing, through SME after restricting the deformation of SMAs and heating them. Due to these properties, SMAs with various chemical compositions have been developed to date and are used in a variety of fields, including aerospace, medical, and automotive [5,6,7]. Among them, Ni-Ti-based SMAs are widely used due to their excellent SME, but their use as construction materials is limited due to their high cost. On the other hand, Fe-based SMAs have a wide hysteresis of transformation, excellent workability, and thermomechanical properties, and can be utilized in construction materials such as structural reinforcement, rail couplings, and pipe joints due to their relatively low cost compared to Ni-Ti-based SMAs [8,9,10]. These advantages suggest that Fe-based SMAs can be a substitute for Ni-Ti-based SMAs in fields requiring high SME.

Sato et al. [11] reported that SME can also be expressed in Fe-Mn-Si SMA. Subsequently, Fe-Mn-Si-based SMAs with various chemical compositions were studied and developed. The early Fe-Mn-Si-based SMAs showed lower SME than Ni-Ti-SMAs. The thermomechanical training, which was performed to improve the SME of Fe-Mn-Si-based SMAs, was reported to increase the recovery strain from 1% to more than 3% [12]. However, the use of Fe-Mn-Si-based SMAs was limited despite their excellent SME because thermomechanical training increases the processing cost compared to the low cost of the raw materials. Dong et al. [13] demonstrated that Fe-Mn-Si-based SMAs with VC precipitates can recover 70% of their shape after 4% deformation without heat treatment. Some researchers have developed Fe-17Mn-5Si-10Cr-4Ni-1(V,C) SMAs without heat treatment that have high strength, ductility, and SME, which have shown potential as prestressing members in the construction industry [14,15]. Several studies have been conducted to apply Fe-17Mn-5Si-10Cr-4Ni-1(V,C) SMAs as reinforcement for bending [16,17,18], shear [19,20], and columns [21,22], and it has been demonstrated that the recovery stress applied through SMAs is effective in improving structural performance. While the fatigue behavior of Fe-Mn-Si-based SMAs has been extensively studied, research specifically focused on the fatigue behavior of welded Fe-Mn-Si-based SMAs remains relatively scarce [23,24,25,26].

Cyclic loading-induced fatigue failure occurs at relatively low stress levels due to the accumulation of localized plastic deformation and residual stress [27]. SMAs exhibit reduced fatigue performance due to the irreversible nature of the transformation caused by plastic deformation of the austenite-martensite phase. In particular, Fe-based SMAs have been reported to exhibit lower fatigue performance than SMAs with other compositions [28,29]. Some researchers have conducted studies to investigate the fatigue behavior of Fe-Mn-Si SMAs. Koster et al. [30] conducted fatigue experiments using the cyclic stress control method to evaluate the fatigue behavior of Fe-Mn-Si SMAs. They confirmed that Fe-Mn-Si SMAs have a fatigue strength of 450 MPa. They also investigated the fatigue behavior by examining the microstructure and confirmed that fatigue damage occurs in the form of accumulation of localized plastic deformation. Ghafoori et al. [31] conducted experimental studies to investigate the fatigue behavior of Fe-Mn-Si SMAs using the cyclic displacement control method. They reported that fatigue failure did not occur in Fe-Mn-Si SMAs at cyclic displacement amplitudes of 0.035% and 0.077%. They also confirmed that fatigue failure occurs due to the accumulation of plastic deformation, which is consistent with the results of Koster et al. [30]. Marinopoulou and Katakalos [32] experimentally investigated the fatigue behavior of Fe-Mn-Si SMAs under low-cycle fatigue loading. They conducted low-cycle fatigue tests considering four load cycles (0.5, 1, 2, and 4 Hz). They proved that the recovery stress can be maintained at a level of about 97% to 98% after low-cycle fatigue testing.

Previous studies conducted by [30,31,32] have provided an understanding of the fatigue behavior of Fe-Mn-Si SMA under various loading conditions. However, these studies primarily focused on unwelded Fe-Mn-Si SMA, and the impact of welding on fatigue behavior was scarcely investigated. Although research has been conducted on the fatigue behavior of Fe-Mn-Si SMAs by some researchers, to the best of the author’s knowledge, no studies have been reported on the fatigue behavior of welded Fe-Mn-Si SMAs. Fatigue failure caused by fatigue loading at welded joints of different materials occurs at relatively low stress levels due to stress concentration between the base material and weld material [33]. The study of the fatigue behavior of welded Fe-Mn-Si SMA is important, considering its potential applications in the construction sector for prestressing, repair and reinforcement tasks. Moreover, the welding applied to the Fe-Mn-Si SMA can alter the microstructure and mechanical properties of the material, significantly impacting its fatigue life. Therefore, investigating the fatigue behavior of welded Fe-Mn-Si SMA is essential not only for predicting fatigue life but also for ensuring the reliability and safety of materials used in the construction field. In addition, welded materials are widely used in construction and infrastructure facilities. This study provides guidance for civil engineering applications where controlling the mechanical properties of welded Fe-Mn-Si SMA is crucial, offering engineering insights particularly in areas subject to cyclic loading, such as structural reinforcements, dampers, and couplings. In particular, when Fe-Mn-Si SMA is used in couplers for pipe joints, it offers a simple yet innovative method of confinement through recovery stress. However, to realize this potential, the effects of welding on the mechanical properties and recovery stress of Fe-Mn-Si SMA must be carefully evaluated, particularly with regard to fatigue performance under sustained hydraulic pressure. Therefore, it is important to investigate the fatigue behavior of welded Fe-Mn-Si SMAs for future use of Fe-Mn-Si SMAs as structural members. Therefore, this paper conducted experimental studies to clarify the fatigue behavior of welded Fe-Mn-Si SMAs. In addition, the surface of the specimen where fatigue failure occurred was analyzed using Scanning Electron Microscopy (SEM, JSM-7200F, JEOL Inc., Tokyo, Japan) to analyze the microstructure of Fe-Mn-Si SMAs. This study is a significant investigation into the potential use of Fe-Mn-Si SMA as a structural material. In particular, it highlights the potential of this material in structural reinforcement, prestressing, and civil engineering applications by elucidating the impact of welding on its mechanical properties and fatigue performance.

## 2. Materials and Methods

### 2.1. Test Material

**Figure 1:**
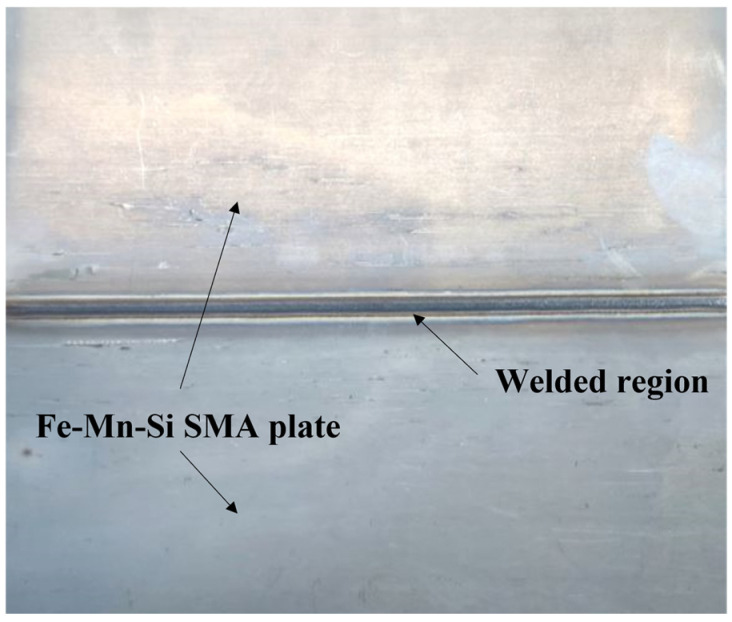
Welded Fe-Mn-Si SMA plate.

In this study, Fe-Mn-Si SMA plates with the chemical composition of Fe-17Mn-5Si-10Cr-4Ni-1(V,C) developed by Empa in Switzerland were used for specimen preparation. Table 1 shows the experimental variables of the Fe-Mn-Si SMA specimens used in the experiment. The experimental variables were welding (C: Control, W: Welding) and heat treatment (N: Non-heat treatment, H: Heat treatment). The specimen SCN, which was not welded and fabricated from a single SMA plate, was fabricated for performance comparison with other specimens. Figure 1 shows the Fe-Mn-Si SMA plate welded for this study, demonstrating the uniform weld achieved using pulsed mode arc welding operating at a pulse frequency of 5 Hz. The thickness and width of the Fe-Mn-Si SMA plate used in this study are 1.55 mm and 120 mm, respectively. The Fe-Mn-Si SMA plate was joined using pulsed mode gas tungsten arc welding (GTAW) operating at a pulse frequency of 5 Hz. In GTAW, a non-consumable tungsten electrode is used to create an arc, which generates the high temperatures necessary to melt and fuse the base material and filler. The filler is an additional material used to fill gaps in the weld joint. In this study, GTAW was performed without using filler, relying solely on the base material. Inert gases such as argon (Ar) and helium (He) are used to protect the molten weld pool. For GTAW, Ar was used as the shielding and purging gas at flow rates of 20 L/min and 10 L/min, respectively. The welding parameters were an arc length of 10 V, torch angle of 10 degrees, current of 100.5 A, and welding speed of 30 cm/min. The welded and heat-treated SWH specimen was obtained by heat-treating the fabricated SWN specimen. The heat treatment conditions were 600 °C for 20 h and 670 °C for 6 h, as proposed by Yang et al. [34]. Yang et al. [35] conducted extensive heat treatment at 600 °C for over 100 h to enhance the density of fine VC precipitates and improve the alloy’s properties. However, such prolonged heat treatment periods may be impractical and inefficient for industrial applications. To optimize the heat treatment process, a two-stage aging treatment was proposed by Yang et al. [34]. The initial stage involves maintaining a high driving force at 600 °C, conducive to strong nucleation of precipitates, facilitating the growth of high-density fine nuclei. This is succeeded by a second stage at an elevated temperature of 670 °C, aimed at promoting the growth of precipitates without the coarsening of nuclei.

### 2.2. Microstructures and Hardness

The microstructure of welded Fe-Mn-Si SMA was investigated using an optical microscope (OM, Eclipse E200, Nikon, Tokyo, Japan). For OM analysis, the welded Fe-Mn-Si SMA was polished with 3 μm and 1 μm alumina suspensions, and 0.04 μm colloidal silica suspension (OP-S NonDry, Struers, Copenhagen, Denmark), and etched with a solution of H_2_O_2_ (35%) (Junsei chemical Co., Ltd., Tokyo, Japan)/HNO_3_ (65%)/HCI (32%) (Samchun Chemicals, Seoul, Republic of Korea) in a ratio of 7/30/9.

The microstructure of the Fe-Mn-Si SMA after welding and post-weld heat treatment was analyzed through SEM scanning electron microscopy (SEM, JSM-7200F, JEOL Inc., Tokyo, Japan), equipped with energy dispersive spectrometry (EDS, X-MaxN, Oxford Instrument, Abingdon, UK). SEM images and EDS element maps were obtained by a backscattered electron mode with an acceleration voltage of 15 kV and a working distance of 6 mm. Before observing the microstructure, all specimens were polished using standard metallographic techniques and etched with a solution of H_2_O_2_ (35%)/HNO_3_ (65%)/HCI (32%) in a ratio of 7/30/9. SEM was used to analyze the microstructure of the fracture surface of specimens subjected to fatigue testing. Before observing the microstructure, all specimens were polished and etched using the same method previously mentioned. In addition, a Vickers hardness test was performed with a load of 300 g and a dwell time of 15 s to measure the hardness of the base metal and weld metal of Fe-Mn-Si SMA.

### 2.3. Mechanical and Recovery Properties

**Figure 2:**
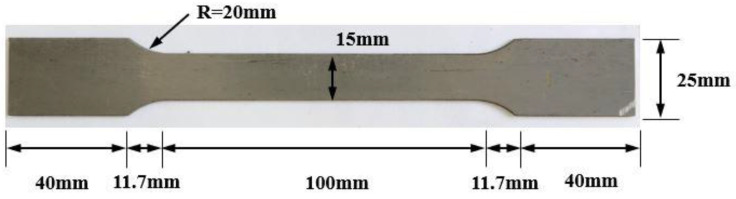
Specimen geometry of direct tensile test.

Figure 2 shows the shape of the direct tensile test specimen specified in ASTM A370 [36], used to evaluate the mechanical properties of the specimens. The specimen measures 203.4 mm in length, 15 mm in width, and 1.55 mm in thickness. A strain gauge was attached to the center of the specimen to measure strain during the direct tensile test. The experiment was conducted using an Instron 8516 (Instron Corporation, Canton, MA, USA) with a capacity of 100 kN, and data were collected at 1 s intervals through a TDS-530 (Tokyo Sokki Kenkyujo Co., Ltd., Tokyo, Japan).

**Figure 3:**
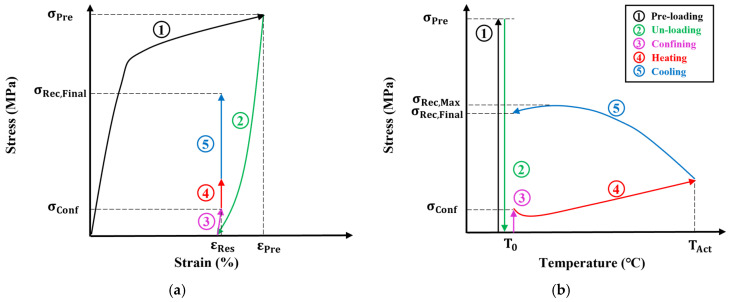
Recovery stress test process of Fe-Mn-Si SMA. (**a**) Stress–strain curve; (**b**) stress–temperature curve.

Recovery stress tests were conducted to investigate the recovery characteristics of welded Fe-Mn-Si SMAs. Figure 3a,b show the step-by-step recovery stress test process of the stress–strain curve and stress–temperature curve, respectively. In the first step of Figure 3, the Fe-Mn-Si SMA is strained to the pre-strain (ε_Pre_). In the second step, the load is removed to generate residual strain (ε_Res_). In the third step, a pre-constrained pressure (σ_Conf_) is applied to prevent thermal expansion due to activation, and the displacement is then constrained. The previous processes are required to be performed before generating the recovery stress of the Fe-Mn-Si SMA. In this study, the same conditions as the recovery stress test of Dong et al. [13], 4% of ε_Pre_ and 50 MPa of σ_Conf_, were applied. In the fourth step, the Fe-Mn-Si SMA is heated from the initial temperature (T_0_) to the activation temperature (T_Act_). In the fifth step, the Fe-Mn-Si SMA is cooled to T_0_. Here, the final recovery stress (σ_Rec,Final_) and the maximum recovery stress (σ_Rec,Max_) of the Fe-Mn-Si SMA due to SME occurs in the 4th and 5th steps, and in particular, the recovery stress is higher in the 5th step than in the 4th step due to thermal contraction.

**Figure 4:**
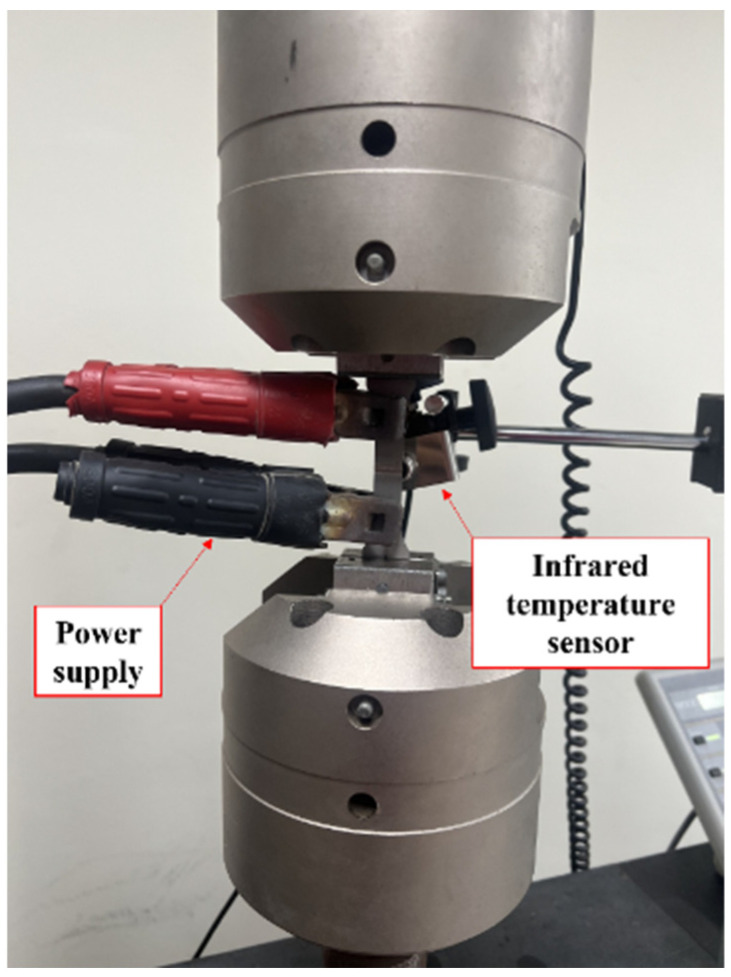
Test setup of recovery stress test.

In this study, recovery stress tests were conducted considering activation temperatures of 160 °C, 200 °C, and 240 °C. Figure 4 shows the test setup for the recovery stress test. The heating of the Fe-Mn-Si SMAs was performed by electrical resistance heating as shown in Figure 4. The surface temperature of the specimen was measured using a non-contact infrared temperature sensor (CT09.10, HEITRONICS, Wiesbaden, Germany).

### 2.4. Fatigue Test Method and Specimens

**Figure 5:**
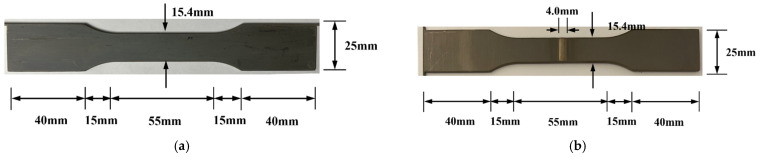
Geometry of fatigue specimens (**a**) SCN; (**b**) SWN.

Specimens were fabricated in accordance with the ASTM E466 [37] standard to investigate the fatigue behavior of welded Fe-Mn-Si SMAs. Figure 5a,b show the SCN and SWN specimens, respectively. The SWH specimen has the same shape as the SWN. All specimens are 1.55 mm thick and 165 mm long. The width of the jig and central part of the specimen are 25 mm and 15.4 mm, respectively. The width of the weld joint of the SWN and SWH specimens is 4 mm, as shown in Figure 5b.

**Figure 6:**
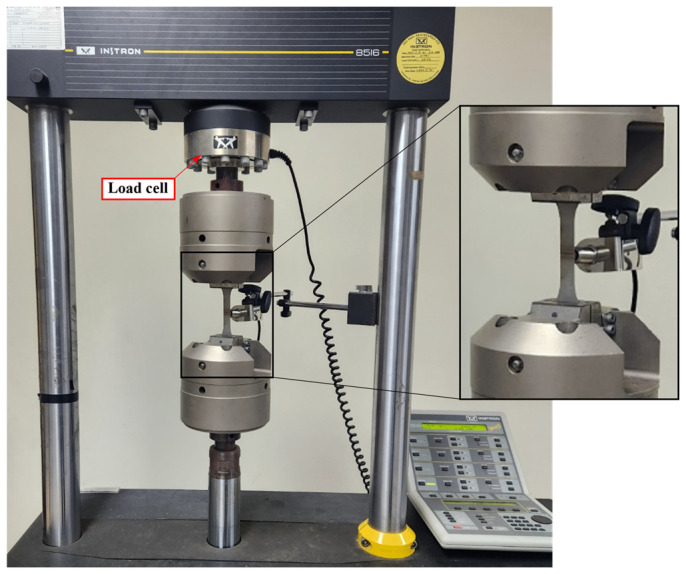
Test setup of fatigue test.

Figure 6 shows the test setup for fatigue testing. The high-cycle fatigue test of the Fe-Mn-Si SMA was conducted at room temperature (25 °C) using a 100 kN capacity Instron 8516 servo-hydraulic materials testing system, with the stress rate set at 5 cycles per second. The load was measured using a 100 kN capacity load cell. 

**Figure 7:**
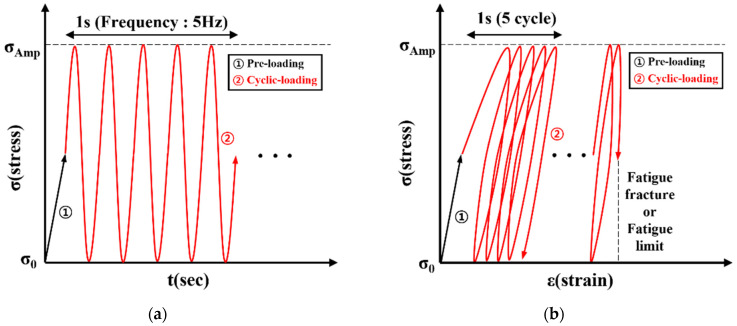
Fatigue test process of Fe-Mn-Si SMA. (**a**) Stress–time curve; (**b**) stress–strain curve.

Figure 7 shows the fatigue testing process of Fe-Mn-Si SMA. All fatigue specimens were preloaded to 50% of the stress amplitude, followed by cyclic loading according to the stress amplitude. All fatigue specimens were subjected to a sinusoidal load between the stress amplitude (σ_Amp_) and the minimum stress (σ_0_) at a frequency of 5 Hz, as shown in Figure 7a. Figure 7b displays the stress–strain curve of the fatigue test, where the test was conducted under the cyclic load at the σ_Amp_ level until fatigue failure or the fatigue limit was reached. The maximum stress amplitude was set to 700 MPa, which is approximately 70% of the ultimate strength, based on the results of direct tensile testing. The minimum stress level was set to 0 MPa. Additionally, if a fatigue fracture occurred before the fatigue limit was reached, the maximum stress level was decreased by 100 MPa. On the other hand, if a fatigue fracture did not occur due to fatigue loading, the average value of the current maximum stress level and the maximum stress level of the previous test was set as the next maximum stress range. Here, the fatigue limit was defined as 2 million cycles of load repetition, according to ASTM E1823 [38]. For the fatigue testing of Fe-Mn-Si SMA specimens, a total of three specimens were tested for each experimental variable to ensure the reliability of the results. The fatigue test results presented are from the specimen that was closest to the average of the three tests.

## 3. Test Results

### 3.1. Microstructural Characterization

**Figure 8:**
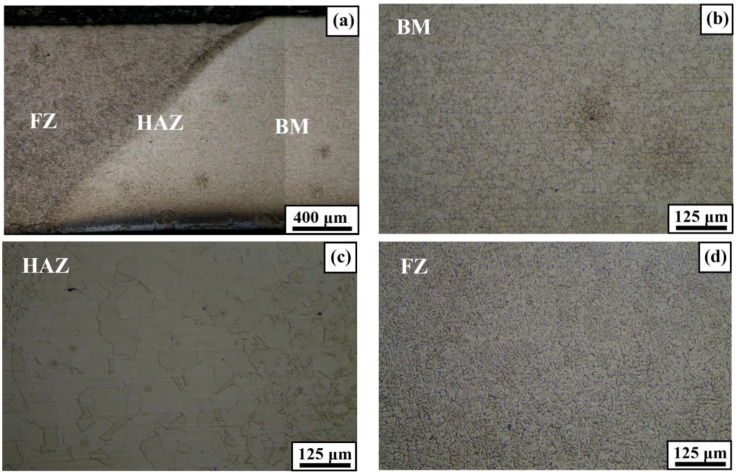
OM image of welded Fe-Mn-Si SMA. (**a**) OM image in different zones; (**b**) highlighted image of BM; (**c**) highlighted image of HAZ; (**d**) highlighted image of FZ.

Figure 8 shows an OM image of the welded Fe-Mn-Si SMA. As shown in Figure 8a, the weldment did not exhibit any welding defects such as pores or cracks along the weld, and distinct regions of the fusion zone (FZ), heat-affected zone (HAZ), and base material (BM) were observed. Figure 8b shows that the BM maintained a microstructure with a uniform grain size even after the welding process. In contrast, Figure 8c shows the OM image of the HAZ in the welded Fe-Mn-Si SMA. Compared to Figure 8a, Figure 8c confirms that grain growth occurred due to the thermal cycles during welding [39]. As the transition occurs from the HAZ to the FZ, the microstructure shifts from a granular structure to dendritic particles in a circumferential arrangement, as shown in Figure 8a. Figure 8d confirms that the FZ comprises dendritic particles in a circumferential arrangement, resulting from remelting during the welding process [40].

**Figure 9:**
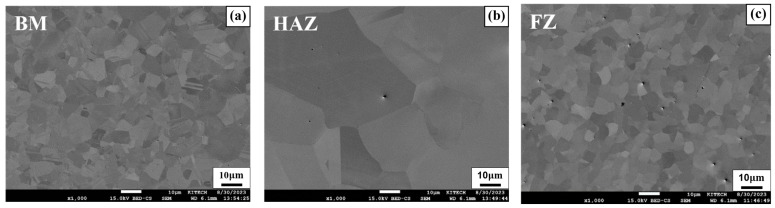
SEM image of SWN (**a**) BM; (**b**) HAZ; (**c**) FZ.

Figure 9a–c show the SEM images of the welded SWN specimen for three different regions (BM, HAZ, FZ), respectively. Figure 9a,c, representing the BM and FZ regions, showed particles and grain boundaries of similar sizes. Notably, horizontal lines caused by the rolling process were observed in the BM region, which did not appear in the HAZ and FZ due to the melting of the alloy during the transition. The HAZ region shown in Figure 9b exhibited a significant increase in grain size compared to the BM and FZ regions, due to the high amount of heat conducted during the welding process. This clearly demonstrates the growth of particles observed in Figure 8c through OM.

**Figure 10:**
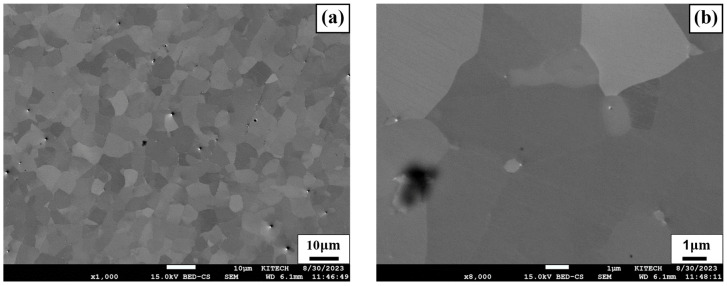

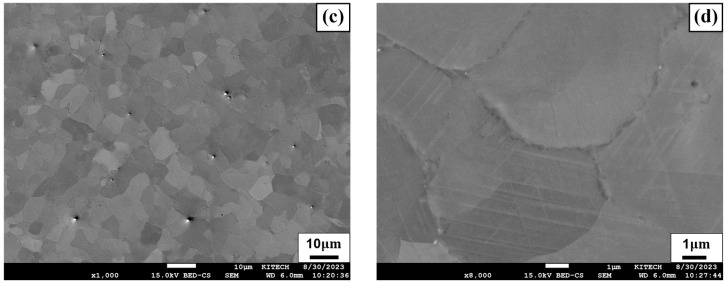
SEM image of FZ before and after heat treatment (**a**) 1000× image of SWN; (**b**) 8000× image of SWN; (**c**) 1000× image of SWH; (**d**) 8000× image of SWH.

Figure 10 shows SEM images of FZ before and after heat treatment. Figure 10a,c show SEM images of SWN and SWH specimens before and after heat treatment, respectively. The grain boundaries appear more prominent after heat treatment compared to the untreated condition. This is confirmed in Figure 10b,d, which show enlarged images of Figure 10a,c, where the SWH specimen forms clear grain boundary precipitates compared to the SWN specimen. Additionally, the white straight lines observed in Figure 10d represent ε-martensite, characterized by a hexagonal close-packed (HCP) crystal structure [41]. The phase transformation from γ-austenite, with a face-centered cubic (FCC) structure, to ε-martensite, with an HCP structure, results from particle-induced precipitation behavior during heat treatment, as described by Kajiwara et al. [42]. Fe-Mn-Si SMA also shows stress-induced martensite due to particle deformation, exhibiting pseudoelastic behavior and recoverable deformation under cyclic fatigue loading. Notably, VC precipitates, induced by heat treatment, facilitate the nucleation of ε-martensite, thereby stabilizing the microstructure under fatigue loading.

**Figure 11:**
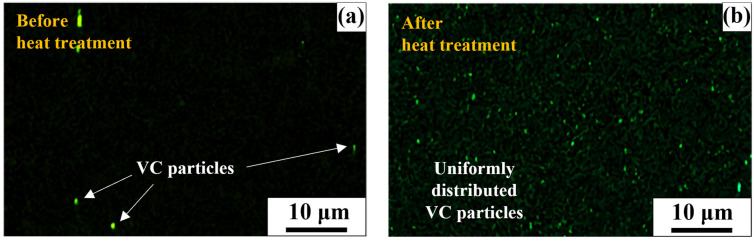
EDS image of VC particles (**a**) SWN; (**b**) SWH.

Figure 11 presents the distribution of VC particles obtained through Electrical Dispersive Spectroscopy (EDS). Figure 11a shows the distribution of VC particles in the non-heat-treated SWN specimen, where it can be observed that the VC precipitates are concentrated rather than uniformly distributed. In contrast, Figure 11b shows the distribution of VC particles in the heat-treated SWH specimen, demonstrating that after heat treatment, the VC precipitates are uniformly distributed both along the grain boundaries and within the grains. Similar observations of VC precipitates distributed both at the grain boundaries and within the grains under the same heat treatment conditions were reported in the study by Yang et al. [34]. The increased density of VC precipitates is advantageous for precipitation hardening, thereby enhancing hardness and strength.

### 3.2. Hardness Distribution

**Figure 12:**
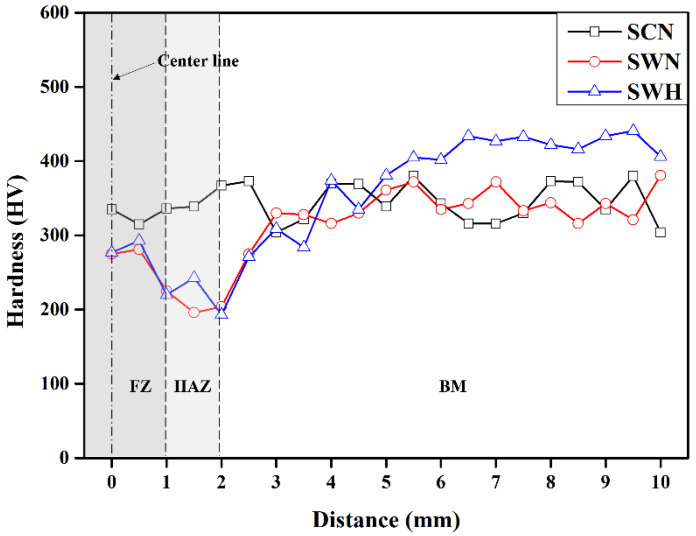
Hardness results of specimens.

Figure 12 shows the hardness results of Fe-Mn-Si SMA at various measurement locations. Hardness was measured at 0.5 mm intervals up to 10 mm from the centerline of the specimen, and the average values of the measured hardness are represented in one direction, as shown in Figure 12. The hardness of the SCN specimen was relatively constant, ranging from 304 HV to 380 HV, as all segments were in the BM region. In contrast, the hardness of the SWN and SWH specimens decreased in the HAZ and FZ regions compared to the BM region. In particular, the hardness of the HAZ transition region of the SWN and SWH specimens decreased by 41.9% and 49.9%, respectively, compared to the average hardness of the BM region. This is consistent with the findings of previous studies by Martins et al. [43] and is attributed to the intrusion of internal precipitates and changes in the crystallographic structure during the welding process. In addition, heat treatment of Fe-Mn-Si SMA increased the hardness in the BM region by 14.3%, but the increase in hardness in the HAZ region was negligible. This is because the hardness increases with increasing strength, as reported by Zhu et al. [44], and the SWH specimen has higher mechanical properties than the SWN specimen. The changes in hardness across different regions (BM, HAZ, FZ) of the welded and post-weld heat-treated Fe-Mn-Si SMA are a result of the microstructural changes induced by welding and heat treatment. As observed in images obtained from OM and SEM, the horizontal lines in the BM, resulting from rolling, likely led to a reduction in the hardening effect due to the melting of the alloy during the welding process. Additionally, the decrease in hardness in the HAZ region can be attributed to the increased grain size in the HAZ, as shown in Figure 8c and Figure 9b. In the case of the FZ region, the relatively lower decrease in hardness was due to the dendritic structure and grain growth resulting from remelting during welding.

### 3.3. Mechanical Properties

**Figure 13:**
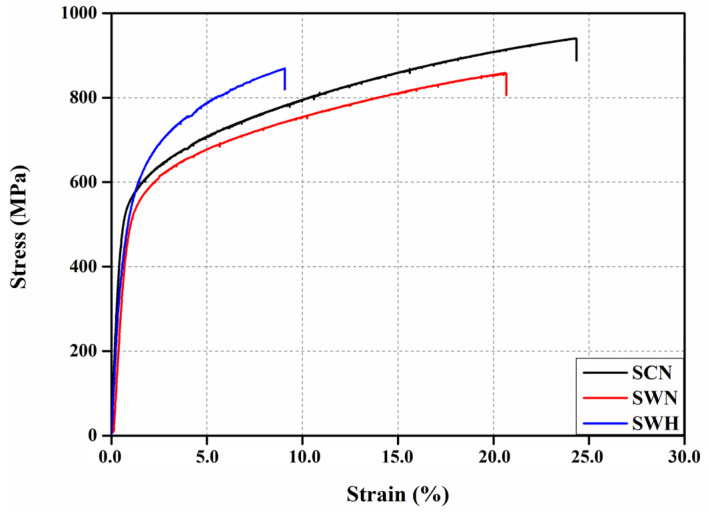
Stress–strain curves of direct tensile test specimens.

Figure 13 presents the stress–strain curves of the SCN, SWN, and SWH specimens obtained through direct tensile testing; mechanical properties for all specimens are summarized in Table 2. The yield strength of Fe-Mn-Si SMA is not clear, as shown in Figure 8. This is due to the stress-induced characteristic caused by the transformation of martensite as the stress of Fe-Mn-Si SMA increases [14,45]. Therefore, in this study, the yield strength of Fe-Mn-Si SMA was defined using the 0.2% offset method. The elastic modulus of the Fe-Mn-Si SMA was calculated through the slope of the stress–strain curve obtained from direct tensile tests within the range of 50 MPa to 200 MPa. The calculated elastic modulus for the SCN, SWN, and SWH specimens were found to be 115.5 GPa, 74.6 GPa, and 88.5 GPa, respectively. The elastic modulus of the SWN specimen decreased by 35.4% compared to the SCN specimen as it was welded, and the elastic modulus of the SWH specimen after welding and heat treatment increased by 18.6% compared to the SWN specimen. This reduction in mechanical properties is attributed to grain coarsening and the associated decrease in hardness, as confirmed by hardness measurements in the HAZ region during the thermal cycle of welding applied to Fe-Mn-Si SMA, as shown in Figure 9b and Figure 12 [43,46]. In contrast, the SCN specimens exhibit a higher modulus of elasticity due to the rolling effect and the relatively small and uniform grain size in the BM region as shown in Figure 9a. Furthermore, the yield strength and ultimate strength of the SWH specimens increased by 4.89% and 1.26%, respectively, compared to the SWN specimens. On the other hand, the elongation of SWN and SWH specimens decreased by 14.8% and 62.6%, respectively, compared to SCN. The increase and decrease in strength and elongation due to heat treatment mitigate the loss of strength due to precipitation hardening from VC precipitates during heat treatment, as shown in Figure 11. However, precipitation hardening during the heat treatment process impedes dislocation movement, and particularly, the elongation of the SWH specimens after welding and heat treatment is lower than that of the SWN specimens due to the increased stiffness caused by precipitation hardening during the heat treatment process [47].

### 3.4. Recovery Properties

**Figure 14:**
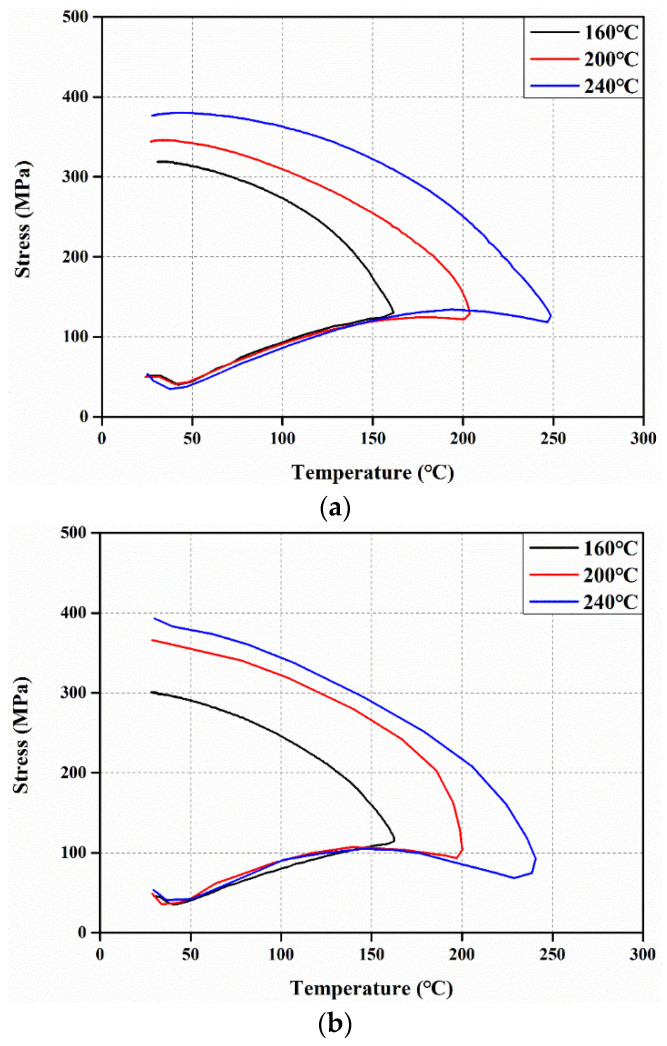

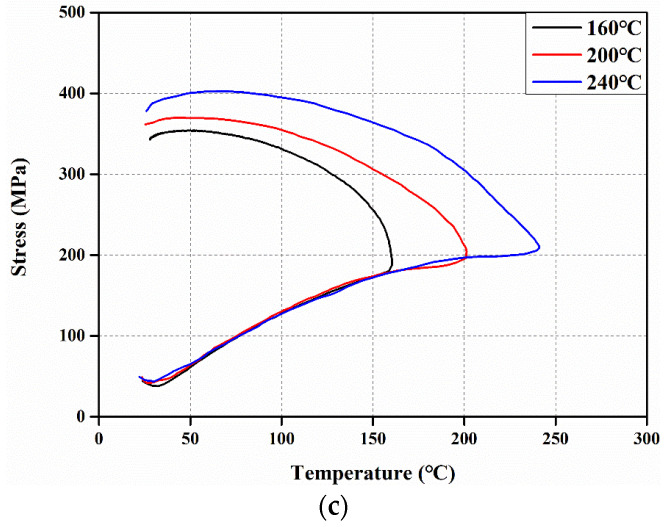
Stress–temperature curves of Fe-Mn-Si SMA (**a**) SCN; (**b**) SWN; (**c**) SWH.

Figure 14 shows the stress–temperature curves of Fe-Mn-Si SMAs measured through recovery stress tests. The recovery stress of Fe-Mn-Si SMAs is presented in Table 3. Here, σ_Rec,Max_ represents the maximum recovery stress measured during the heating process of the Fe-Mn-Si SMA specimen, while σ_Rec,Final_ denotes the recovery stress measured when the Fe-Mn-Si SMA specimen is cooled back to the initial temperature (T_0_) after heating. The stress reduction due to thermal expansion occurred up to about 40 °C for all specimens, and the stress decreased by an average of 20.2% compared to σ_Conf_. Figure 14a,c show the stress–temperature curves of SCN and SWH specimens, and the recovery stress increased during the heating and cooling processes. On the other hand, the recovery stress of the SWN specimen showed a decreasing trend until it reached the maximum temperature at a heating temperature of more than 200 °C, as shown in Figure 14b. The subsequent sharp increase in recovery stress of Fe-Mn-Si SMA occurred between the austenite start temperature (A_s_) of 103 °C and the austenite finish temperature (A_f_) of 163 °C, as reported by Shahverdi et al. [48]. Notably, as the Fe-Mn-Si SMA specimen reached the austenite A_f_, the increase in recovery stress was observed to slow down. This is because, beyond the austenite A_f_, the reverse transformation of ε-martensite is no longer induced. Additionally, the specimen exhibited an increasing trend in recovery stress during the cooling process, which is attributed to the increase in both elastic modulus and recovery stress caused by the thermally induced martensitic transformation during cooling [49]. The difference between σ_Rec,Max_ and the σ_Rec,Final_ of SCN and SWN was 1.22% on average, which is insignificant, while the recovery stress of SWH decreased by an average of 4.4% at σ_Rec,Final_ compared to σ_Rec,Max_. This is because the SWH specimen, which was hardened by precipitation due to activation, increased in stress and underwent a rapid phase transformation from austenite to martensite as the temperature decreased. When the heating temperature of Fe-Mn-Si SMA increased by 40 °C, the recovery stress of all specimens increased by an average of 10%. This trend occurs because, as the heating temperature increases, ε-martensite fully transforms into γ-austenite, resulting in an increase in recovery stress. This is consistent with the findings of Gu et al. [50], who investigated the recovery stress in relation to heating temperature.

### 3.5. Fracture Location of Fatigue Specimens

**Figure 15:**
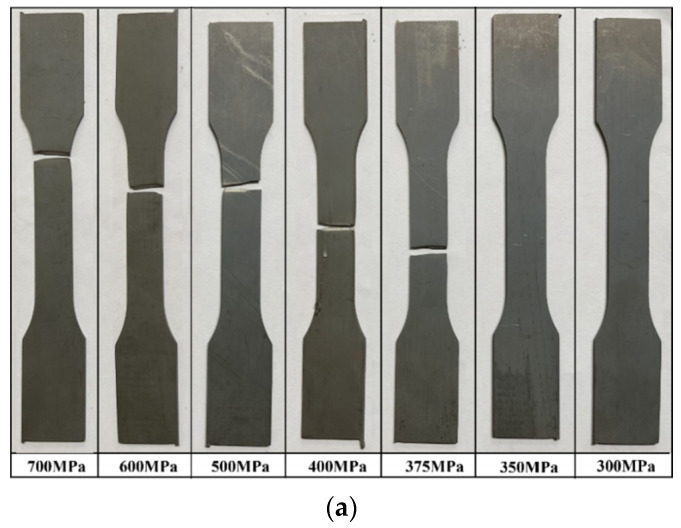

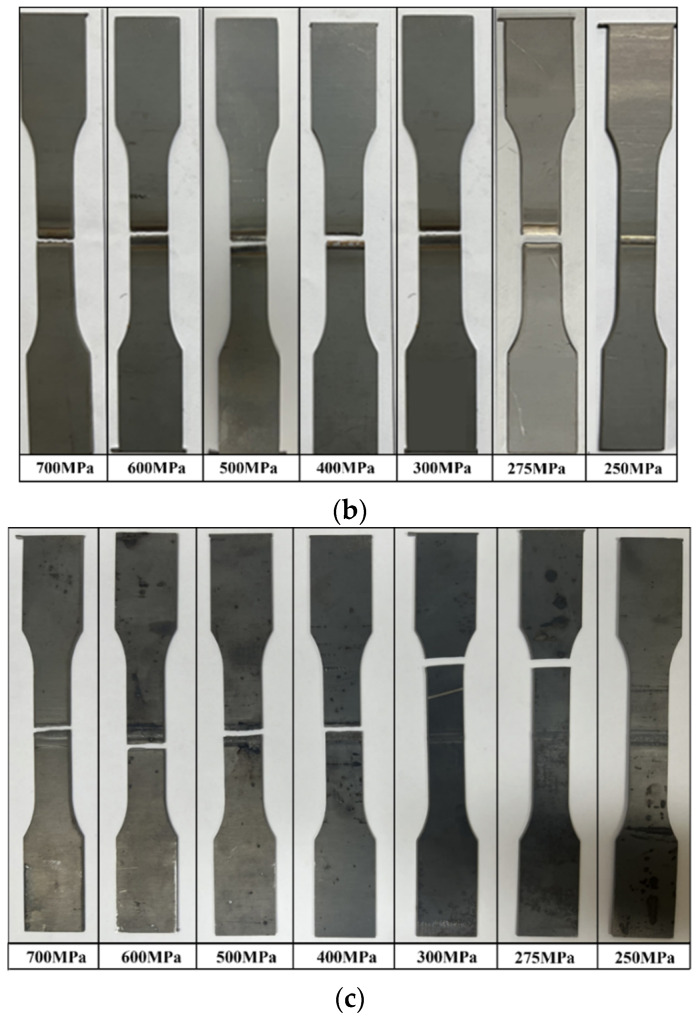
Specimens after fatigue test (**a**) SCN; (**b**) SWN; (**c**) SWH.

**Figure 16:**
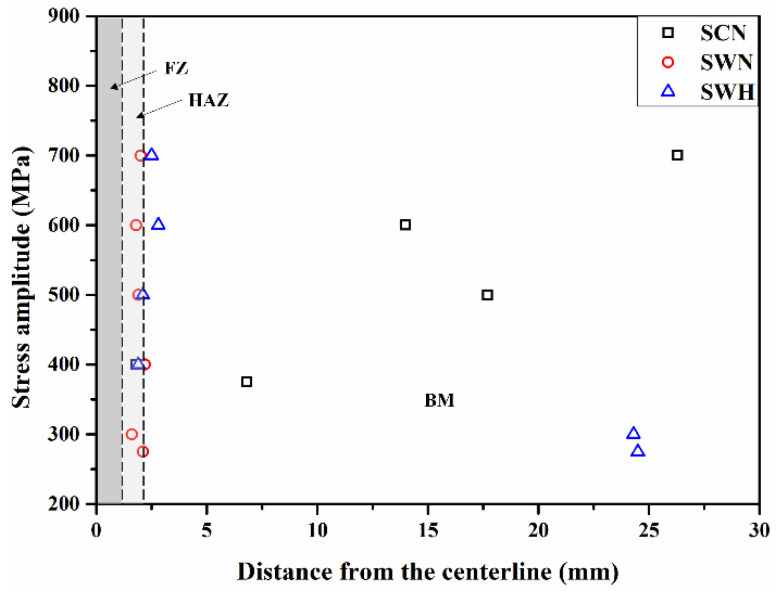
Distribution of fracture location of specimens.

Figure 15 shows the shapes of all specimens after fatigue testing. Figure 15a shows the fracture morphology of the SCN specimen at different stress amplitudes. Fatigue failure of the specimen occurred in the stress range of 375–700 MPa. On the other hand, fatigue failure did not occur even after 2 million cycles at stress amplitudes of 350 MPa or less. Figure 15b,c show the SWN and SWH specimens, respectively. Fatigue failure did not occur at a stress amplitude of 250 MPa. Figure 16 shows the distribution of fracture distances of the SCN, SWN, and SWH specimens at different stress amplitudes. The fracture distance of a specimen is the distance from the center of the specimen to the fracture surface. All fracture distances of the SCN specimen were located in the BM region. The fracture distance tended to move away from the center of the specimen as the stress amplitude increased. On the other hand, the fracture distances of the SWN and SWH specimens, which were welded and post-welded heat-treated, occurred in the HAZ regions. In particular, fracture of the SWH specimen occurred in the BM region at stress amplitudes of 300 MPa or less. The fracture distance tended to move away from the center of the specimen as the stress range decreased.

### 3.6. Stress–Strain Behavior under Cyclic Loading

Table 4 shows the fatigue test results of all specimens at different stress amplitudes. The SCN specimen did not fail under the imposed limit cycles at stress amplitudes below 350 MPa. Similarly, the SWN and SWH specimens did not fail even after exceeding the imposed limit cycles at a stress amplitude of 250 MPa. All specimens exhibited high-cycle fatigue behavior with a cycle number of more than 10^4^ even at stresses exceeding the yield strength. High-cycle fatigue failure, characterized by cycles exceeding 10^4^, typically occurs at relatively low stress levels within the elastic region, whereas low-cycle fatigue failure, generally occurring within cycles below 10^4^, involves high stress levels that encompass both elastic and plastic deformation. SWN and SWH specimens showed lower cycle numbers at the same stress amplitude than SCN specimens, indicating that fatigue performance deteriorated due to welding and post-welding heat treatment. The SWH specimens showed higher fatigue performance than SWN specimens at high stress amplitudes of 500–700 MPa. However, SWH specimens failed due to fatigue at lower stress amplitudes of 400 MPa or less, with a lower cycle number than SWN specimens. The difference in fatigue performance due to heat treatment is attributed to the fact that a more uniform distribution of VC particles induced by heat treatment resists higher fatigue strength, as reported by Hajisafari et al. [51]. Furthermore, the EDS images in Figure 11 confirm a more uniform distribution of VC particles in the specimens subjected to heat treatment. However, precipitates formed during the heat treatment process are destroyed as the cycle number increases at low stress amplitudes, resulting in a decrease in fatigue resistance at low stress amplitudes.

**Figure 17:**
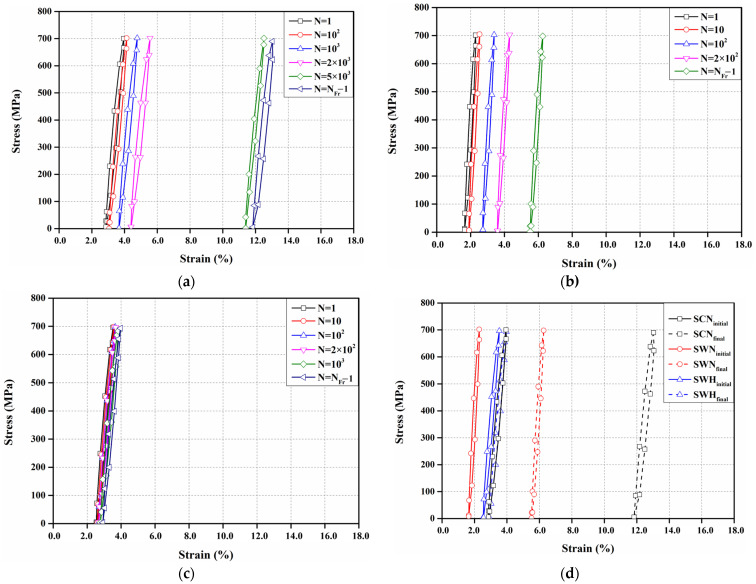
Stress–strain curves according to the number of cycles under stress amplitude of 700 MPa (**a**) SCN; (**b**) SWN; (**c**) SWH; (**d**) initial and final curves.

Figure 17 shows the stress–strain curves of all specimens for a cycle number at a stress amplitude of 700 MPa. The last stress–strain curves of all specimens shown in Figure 17a–c represent the results of the stress–strain curves with one cycle subtracted from the fracture cycle (N_Fr_) of the specimen. The strains of SCN, SWN, and SWH specimens increased, as shown in Figure 17a–c, as the plastic deformation accumulated. Figure 17a shows the stress–strain curve of the SCN specimen for a cycle number, and a significant increase in strain was observed as the cycle number increased from 2 × 10^3^ to 5 × 10^3^. In the case of SWN and SWH specimens, as shown in Figure 17b,c, the strain increased proportionally as the cycle number increased, and the fracture occurred at a relatively lower cycle number than SCN specimen. This is because, as shown in Figure 9, from the microstructure observations, the growth of HAZ grains due to welding reduces the number of particle boundaries that can block dislocation movement, allowing cracks to propagate more easily. As a result, the fracture locations in the SWN and SWH specimens are concentrated in the HAZ. Figure 17d compares the initial and final stress–strain curves for all specimens. The difference in maximum strain between the first and final cycles for the SCN, SWN, and SWH specimens was 9.11%, 3.96%, and 0.43%, respectively, with the SCN specimen showing the greatest deformation. In contrast, although the SWH specimen underwent 3760 more fatigue cycles than the SWN specimen, the difference in strain for the SWH specimen was 89.1% lower than that of the SWN specimen. This indicates that the SWH specimen has higher strength than the SWN specimen and experiences less deformation under high stress amplitudes.

**Figure 18:**
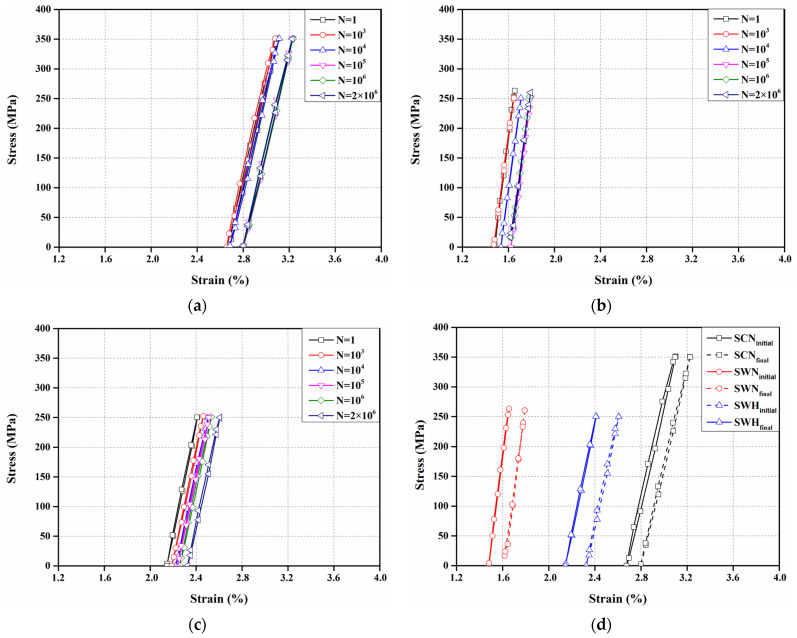
Stress–strain curves according to the number of cycles of specimens without fatigue fracture (**a**) SCN (350 MPa); (**b**) SWN (250 MPa); (**c**) SWH (250 MPa); (**d**) Initial and final curves.

Figure 18 shows the stress–strain curves of all specimens for a cycle number at a stress amplitude that does not cause fractures. The stress–strain curves of all specimens showed an increasing trend in strain as the cycle number increased, as shown in Figure 18a–c. However, the stress–strain curve of the SWN specimen showed a decreasing trend in strain as the cycle number increased to 10^5^, as shown in Figure 18b. This is the same trend as the fatigue test results of Hong et al. [52], and it is because the residual deformation that occurred during the rolling process of manufacturing Fe-Mn-Si SMA is recovered. Figure 18d compares the stress–strain curves of the SCN, SWN, and SWH specimens at the first cycle and at 2 million cycles. The difference in maximum strain between the first and final cycles for the SCN, SWN, and SWH specimens was 0.13%, 0.14%, and 0.20%, respectively. The SCN specimen showed the smallest difference in strain, which is attributed to the higher initial strain due to the higher stress amplitude compared to the SWN and SWH specimens. In contrast, the SWH specimen exhibited a 44% increase in strain difference despite being subjected to the same stress amplitude as the SWN specimen. This is a different trend from the results obtained at a stress amplitude of 700 MPa. The precipitates that occurred during the heat treatment of the SWH specimen decreased the fatigue performance as the cycle number increased, leading to an increase in plastic deformation.

### 3.7. Fatigue Life

**Figure 19:**
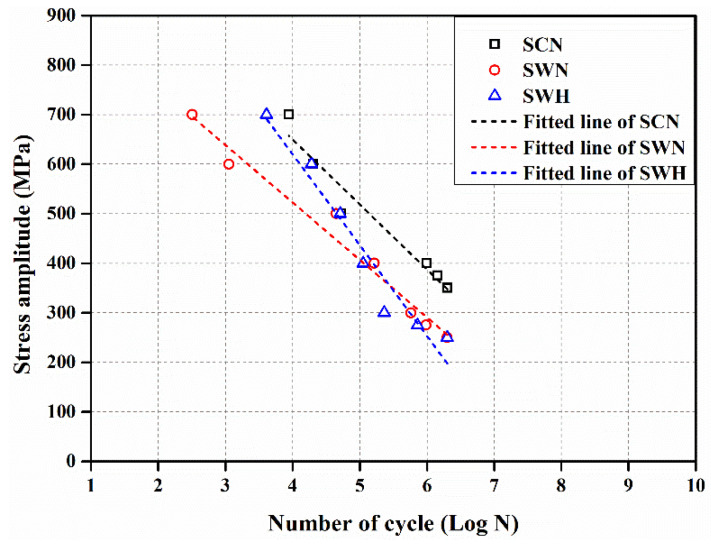
S-N curve of Fe-Mn-Si SMA.

Figure 19 shows the stress amplitude–cycle number curves and the results of linear regression analysis for all specimens. Fatigue life increased as stress amplitude decreased for all specimens. Fatigue life of Fe-Mn-Si SMA was found to decrease for the SWN and SWH specimens compared to the SCN specimen. This is attributed to stress-induced transformation from austenite to martensite under high fatigue loads, as reported by Sawaguchi et al. [53]. The linear regression analysis results curves for the SCN and SWN specimens showed similar slopes, as shown in Figure 19. This is because fatigue performance was partially degraded by welding in Fe-Mn-Si SMA, but the stress–strain behavior of the specimen material is similar, as shown in Figure 13. The linear regression analysis results curve for the SWH specimen showed a steeper slope than the SCN and SWN specimens. Therefore, it was confirmed that the heat treatment applied to welded Fe-Mn-Si SMA can improve fatigue life above yield stress, but it can reduce fatigue performance below yield stress.

Equations (1)–(3) are the equations obtained by linear regression analysis of the experimental results of SCN, SWN, and SWH specimens.

(1) $$\sigma_{SCN}=-131.22logN+1174.8$$

(2) $$\sigma_{SWN}=-115.96logN+986.51$$

(3) $$\sigma_{SWH}=-183.46logN+1353.9$$

Here, N is the number of fatigue cycles, and σ_SCN_, σ_SWN_ and σ_SWH_ are the fatigue limit values corresponding to the fatigue life. The determination coefficients of the experimental results and Equations (1)–(3) were 0.947, 0.973, and 0.947, respectively, indicating a high correlation. The fatigue strengths of the SCN, SWN, and SWH specimens calculated by substituting 2 million cycles, which is assumed to be the number of cycles at which fatigue failure does not occur in this study, into Equations (1)–(3) were 348.0 MPa, 255.8 MPa, and 197.9 MPa, respectively.

### 3.8. Microstructures of Fatigue Specimens

**Figure 20:**
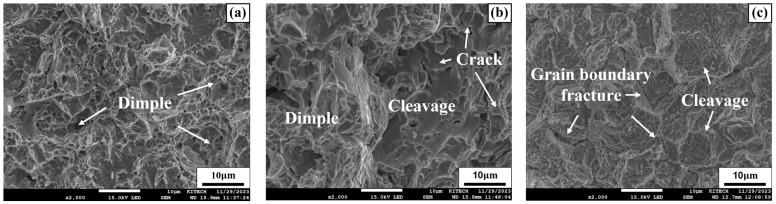
SEM image of specimens at low stress amplitude (**a**) SCN (400 MPa); (**b**) SWN (300 MPa); (**c**) SWH (300 MPa).

The fracture surfaces of the specimens after fatigue testing were analyzed using SEM. Figure 20a–c show SEM images of SCN, SWN, and SWH specimens at low stress amplitudes. Figure 20a shows the fracture surface of the SCN specimen, with cup-shaped dimples observed. The presence of dimples on the fracture surface of an alloy is a major characteristic of ductile fracture mode. In Figure 20b, cleavage river patterns, which are secondary cracks occurring on the plane of the crystal structure, were observed along with the same dimples observed in Figure 20a. The formation of secondary cracks can relieve the stress at the crack tip, allowing it to absorb more energy and slow down the propagation of fatigue cracks [54]. On the other hand, grain boundary fracture due to cracks propagating along grain boundaries was observed as the main failure mode in the SWH specimen, as shown in Figure 20c. The poor fatigue performance of SWN and SWH specimens at low stress amplitudes is attributed to the inhomogeneous microstructure due to the presence of many precipitates in the dendrite structure of the weld. In addition, the poor fatigue performance of the SWH specimen compared to the SWN specimen is due to intergranular brittle fracture caused by the detachment of precipitates from the grain boundaries, accelerating the growth of the crack, as shown in Figure 20c.

**Figure 21:**
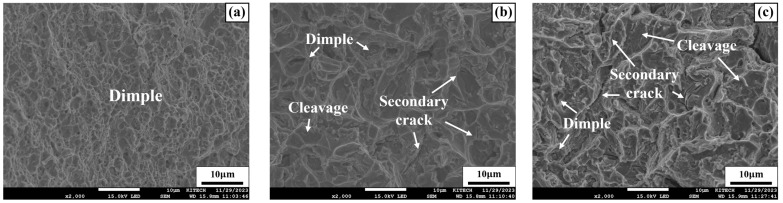
SEM image of specimens at high stress amplitude (**a**) SCN (700 MPa); (**b**) SWN (700 MPa); (**c**) SWH (700 MPa).

Figure 21a–c show the SEM images of SCN, SWN, and SWH specimens under high stress amplitude of 700 MPa. Figure 21a shows the fracture surface of the SCN specimen, which exhibits typical ductile fracture due to dimple rupture. On the other hand, Figure 21b,c show the fracture surfaces of the SWN and SWH specimens, respectively. The fracture modes of these specimens are a mixture of dimples and cleavage, with fewer dimples than the SCN specimen. Additionally, significant secondary cracks are observed on the fracture surfaces, indicating brittle fracture. However, the fatigue performance at high stress amplitude was higher in the SWH specimen than in the SWN specimen, unlike the results at low stress amplitude. This is because high stress amplitude fatigue fracture is significantly affected by the strength of the material, as reported by Grönlund et al. [55]. The SWH specimen, which underwent precipitation hardening through heat treatment, has higher tensile strength than the SWN specimen.

## 4. Discussion

In this study, the fatigue behavior of welded Fe-Mn-Si SMA under different stress amplitudes was investigated. Fatigue test results revealed various fracture phenomena occurring in the Fe-Mn-Si SMA depending on the applied stress amplitude, welding, and post-weld heat treatment. Under fatigue loading, 83.3% of the welded Fe-Mn-Si SMA specimens fractured in the HAZ. Microstructural observation indicated that the high mismatch of grain boundary energies between BM and HAZ, due to the high intergranular energy generated, reduces the deformation energy, diminishing the energy available to withstand dynamic loads [56]. Moreover, the high intergranular energy under fatigue loading restricts dislocation mobility, leading to dislocation pile-up. This piled-up dislocation causes stress concentration in local areas, degrading the material fatigue performance. Additionally, the uniform distribution of VC precipitates in the welded and heat-treated Fe-Mn-Si SMA hinders dislocation movement within the crystal structure, increasing resistance to deformation. This can act as a crack initiation due to internal stress concentration under fatigue load. For this reason, the main locations of fatigue fracture appear in the coarse-grained areas of the welded Fe-Mn-Si SMA. Particularly, at high stress amplitudes, the increase in fatigue cycles causes a rapid stress concentration along the grain boundaries, displaying quasi-brittle fatigue behavior with faster fatigue crack growth compared to lower stress amplitudes. Conversely, precipitation hardening by VC precipitates at low stress amplitudes effectively inhibits fatigue crack growth, enhancing fatigue performance. Consequently, the fatigue fracture mechanism of Fe-Mn-Si SMA under various stress amplitudes is linked to the microstructural characteristics of the alloy, especially the formation and distribution of VC precipitates. Understanding these microstructural features and their interaction under cyclic loading conditions is important to assess the effect of stress amplitude on fatigue performance. It is deemed crucial to optimize post-weld heat treatment to enhance the fatigue strength and life of Fe-Mn-Si SMA. Additionally, by analyzing the effects of welding and post-weld heat treatment on the fatigue life of Fe-Mn-Si SMA, this study provides essential foundational data to ensure the reliability and safety of this material in the construction field.

## 5. Conclusions

In this study, the fatigue behavior of welded Fe-Mn-Si SMAs was evaluated, and the following conclusions were drawn based on the experimental results.

1. Welding has been shown to degrade the mechanical properties of Fe-Mn-Si SMA, including a significant reduction in elastic modulus, yield strength, and tensile strength. However, post-weld heat treatment can effectively improve these properties due to precipitation hardening in Fe-Mn-Si SMA, suggesting its potential as a construction material for prestressed applications.
2. The hardness of the heat-affected zone (HAZ) decreased by 41.9% and 49.9% compared to the base metal (BM) and fusion zone (FZ), respectively, due to grain growth and other microstructural changes caused by welding. Conversely, heat treatment increased the hardness of the BM by an average of 14.3%, indicating that controlled heat treatment processes can restore and enhance the material’s hardness.
3. The fatigue test results showed that no low-cycle fatigue failure occurred at stress amplitudes at the yield strength level, and under the fatigue limit condition of 2 million cycles, the fatigue strengths of the SCN, SWN, and SWH specimens were 350 MPa, 250 MPa, and 250 MPa, respectively. Additionally, the differences in fatigue performance between the specimens were primarily due to microstructural changes caused by welding and heat treatment, with heat treatment notably improving fatigue performance at high stress amplitudes.
4. At low stress amplitudes, increasing the stress amplitude caused the fracture mode to transition from ductile dimpling to brittle cleavage. In particular, the particle inhomogeneity in the welded and heat-treated specimens was found to have a negative impact on fatigue performance by creating stress concentrations that facilitate crack initiation.
5. The fatigue results obtained in this study suggest that welded Fe-Mn-Si SMA could achieve excellent fatigue performance in construction components subjected to stress amplitudes below the yield stress level. Additionally, it is necessary to optimize the heat treatment conditions to minimize grain growth and improve particle homogeneity in welded Fe-Mn-Si SMA to further enhance fatigue performance.

## Figures and Tables

**Table 1:** Fe-Mn-Si SMA specimens used in the experiment.

Specimens	Chemical Composition (Weight %)	Welding	Heat Treatment
SCN	Fe-17Mn-5Si-10Cr-4Ni-1(V,C)	Non-welding	Non-heat treatment
SWN		Welding	Non-heat treatment
SWH		Welding	Heat treatment

**Table 2:** Mechanical properties of direct tensile test specimens.

Specimens	Elastic Modulus (GPa)	Yield Strength (MPa)	Ultimate Strength (MPa)	Elongation (%)
SCN	115.5	495.5	940.0	24.3
SWN	74.6	435.7	858.8	20.7
SWH	88.5	457.0	869.6	9.1

**Table 3:** Recovery stress results.

Temperature (°C)	SCN		SWN		SWH	
	σ_Rec,Max_ (MPa)	σ_Rec,Final_ (MPa)	σ_Rec,Max_ (MPa)	σ_Rec,Final_ (MPa)	σ_Rec,Max_ (MPa)	σ_Rec,Final_ (MPa)
160	319.5	318.6	301.22	301.1	354.3	343.0
200	346.1	344.0	365.9	356.9	372.5	360.6
240	380.4	376.8	392.8	392.8	404.3	376.4

**Table 4:** Fatigue test results according to stress amplitude.

Stress Amplitude (MPa)	SCN		SWN		SWH	
	Number of Cycle	Log N	Number of Cycle	Log N	Number of Cycle	Log N
700	8794	3.944	321	2.507	4081	3.611
600	20,363	4.309	1123	3.050	19,041	4.280
500	52,771	4.722	44,440	4.648	50,994	4.708
400	985,858	5.994	164,466	5.216	112,361	5.051
375	1,427,544	6.155	-	-	-	-
350	IL	-	-	-	-	-
300	IL	-	577,574	5.762	230,425	5.363
275	-	-	960,702	5.983	719,431	5.857
250	-	-	IL	-	IL	-

IL: Imposed limit.

## Data Availability

The original contributions presented in the study are included in the article, further inquiries can be directed to the corresponding author.
